# Gengnianchun, a Traditional Chinese Medicine, Enhances Oxidative Stress Resistance and Lifespan in* Caenorhabditis elegans by Modulating* daf-16/FOXO

**DOI:** 10.1155/2017/8432306

**Published:** 2017-03-16

**Authors:** Fanhui Meng, Jun Li, Wenjun Wang, Yan Fu

**Affiliations:** ^1^Department of Gynecology, The First Hospital of Jilin University, Changchun, China; ^2^Department of Integrated Traditional Chinese Medicine and Western Medicine, Obstetrical and Gynecological Hospital, Fudan University, Shanghai, China

## Abstract

*Objective.* Gengnianchun (GNC), a traditional Chinese medicine (TCM), is primarily used to improve declining functions related to aging. In this study, we investigated its prolongevity and stress resistance properties and explored the associated regulatory mechanism using a* Caenorhabditis elegans* model.* Methods.* Wild-type* C. elegans* N2 was used for lifespan analysis and oxidative stress resistance assays. Transgenic animals were used to investigate pathways associated with antioxidative stress activity. The effects of GNC on levels of reactive oxygen species (ROS) and expression of specific genes were examined.* Results.* GNC-treated wild-type worms showed an increase in survival time under both normal and oxidative stress conditions. GNC decreased intracellular ROS levels by 67.95%. GNC significantly enhanced the oxidative stress resistance of several mutant strains, suggesting that the protective effect of GNC is independent of the function of these genes. However, the oxidative stress resistance effect of GNC was absent in worms with daf-16 mutation. We also found upregulation of daf-16 downstream targets including sod-3 and mtl-1.* Conclusions.* Our findings suggest that GNC extends the lifespan of C. elegans and enhances its resistance to oxidative stress via a daf-16/FOXO-dependent pathway. This study also provides a feasible method for screening the biological mechanisms of TCMs.

## 1. Introduction

Gengnianchun (GNC) is a traditional Chinese medicine (TCM) composed of 12 ingredients: Radix Rehmanniae, Rhizoma Coptidis, Radix Paeoniae Alba, Rhizoma Anemarrhenae,* Cistanche salsa*, Radix Morindae Officinalis, Poria,* Epimedium brevicornum*, Cortex Phellodendri Amurensis,* Fructus lycii*, Semen Cuscutae, and Carapax et Plastrum Testudinis. According to TCM theory, GNC has a kidney/liver-tonifying effect that is used to alleviate declining functions related to aging. Moreover, according to clinical data, GNC has therapeutic effects on menopausal symptoms [1]. Recently, numerous studies have indicated that GNC provides an array of beneficial effects. For example, GNC improves learning and memory in ovariectomized rats by increasing hippocampal acetylcholine (ACh), acetylcholinesterase (AChE), and choline acetyltransferase (ChAT) activities [2]. Serum of GNC-treated rats inhibits apoptosis in A*β*-insulted PC-12 cells by regulating Bcl-2 and Bax expression and caspase 3 activation. These effects suggest that GNC may protect against neurodegenerative diseases such as Alzheimer's disease [3]. Furthermore, GNC exerts antiaging effects by modulating the hypothalamus-pituitary-ovary axis, thereby increasing the level of estradiol receptor (ER) in the pituitary gland and ovaries as well as increasing the concentration of *β*-EP in the hypothalamus [4]. More recently, Rao et al. demonstrated that GNC decreased levels of interleukin-1 beta (IL-1*β*), interleukin-6 (IL-6), and tumor necrosis factor-alpha (TNF-*α*) but increased levels of interleukin-2 (IL-2) and interferon-gamma (IFN-*γ*). Using an ovariectomized rat model, GNC was also found to reverse ovariectomy-induced weight gain and leptin resistance, with possible clues regarding the mechanisms through which GNC attenuates age-related diseases [5]. Using a skin-aging model, Yuan demonstrated that GNC significantly increases the concentration of hydroxyproline (HYP) and activity of SOD, suggesting that GNC improves antioxidative defense against aging [6].

Oxidative stress represents an imbalance between toxic reactive oxygen species (ROS) and antioxidant systems. High levels of ROS cause inflammation and cytotoxicity, damaging proteins, lipids, and DNA, and subsequently disrupting cellular functions and resulting in cell death [7]. In humans, oxidative stress plays an important role in many diseases, including cardiovascular diseases [8], neurodegenerative diseases such as Alzheimer's disease [9] and Parkinson's disease [10], autoimmune diseases such as SLE [11], and cancer [12]. Furthermore, numerous studies have demonstrated the extensive involvement of oxidative stress in aging itself [13, 14].

*Caenorhabditis elegans* is a powerful animal model because of its short lifespan, ability to self-fertilize, and ease of culture. Because it possesses genes homologous to two-thirds of those involved in human disease,* C. elegans* is applied as a well-characterized experimental system for studying aging and aging-associated diseases [15], and several studies have shown that enhanced resistance to oxidative stress extends the lifespan of this nematode [16, 17]. In* C. elegans*, stress resistance is related to many factors, including insulin/IGF-1, TOR, and mitochondrial signaling pathways [18–20]. In addition, a rich collection of mutants has been used to explore the molecular mechanisms of the therapeutic components of TCM pharmacopeia, such as Liuwei Dihuang (LWDH) and Aiweixin [21, 22].

To investigate whether the TCM GNC exhibits stress resistance properties, the potential to extend longevity, or both, in vivo lifespan and antioxidant assays were performed. This study also utilized wild-type and mutant* C. elegans* strains to determine associated regulatory mechanisms of the oxidative stress resistance induced by GNC, and the effects of GNC on the expression levels of associated genes were analyzed.

## 2. Materials and Methods

### 2.1. Preparation of the GNC Formula

The GNC formula used contains 12 crude herbs (as shown in Table 1). For this study, we used a mixture of water extracts of the 12 crude herbs. The water extracts, which were purchased from Tianjiang Pharmaceutical (Jiangyin, China), were produced following the rigid specifications of the Pharmacopeia of the People's Republic of China and were CFDA-approved. The conversion between crude herb and water extract is also shown in Table 1. According to conventional TCM research, all concentrations in this study referred to the concentration of the crude herb.

### 2.2. *C. elegans*: Strains and Maintenance

The Caenorhabditis Genetics Center (CGC) at the University of Minnesota (Minneapolis, MN, USA) provided wild-type* C. elegans *N2 (Bristol),* E. coli* OP50, and the following transgenic worms: CB1370, daf-2 (e1370); TJ1052, age-1 (hx546); CF1038, daf-16 (mu86); MQ1333, nuo-6 (qm200); MQ887, isp-1 (qm150); DA465, eat-2 (ad465); RB1206, rsks-1 (ok1255) and CF1903, glp-1 (e2144).* C. elegans* strains were maintained at 20°C on solid nematode growth medium (NGM) plates seeded with* E. coli* OP50. For CB1370, daf-2 (e1370) cultures, the nematodes were maintained at 16°C for 3 days and then transferred to 20°C until the desired stage of development was reached.

### 2.3. Toxicity Test for GNC

The toxic effect of different concentrations of GNC was evaluated. Age-synchronized day 1 adult N2* C. elegans* were incubated with a series of GNC concentrations, from 0.00394 mg/mL to 78.8 mg/mL, in S-complete liquid medium (a liquid culture medium for* C. elegans*) [23]. A final concentration of 400 *μ*M 5-fluoro-2′-deoxyuridine (FUDR, Sigma-Aldrich Co., St. Louis, MO, USA) was added to the medium to block progeny development. Survival was assessed after 48 hours of treatment with GNC. The nematodes were considered dead when they failed to respond to touch using a platinum loop. Ninety worms at each concentration were tested, and the experiment was performed three times independently.

### 2.4. Lifespan Analysis

Age-synchronized day 1 adult N2 nematodes were transferred to a 96-well plate with 1-2 worms in 80 *μ*L of S-complete liquid medium containing various GNC drug concentrations or a vehicle control (H_2_O) in each well.* E. coli* OP50 and FUDR were added to the medium. Survival was assessed every other day until death using the touch-provoked method described above. The lifespan assay was repeated in three independent trails.

### 2.5. Oxidative Stress Resistance Assay

The oxidative stress resistance assay was based on previously published studies [22, 24]. Briefly, age-synchronized day 1 adult worms were incubated in several concentrations of GNC (0.0394, 0.394, 1.97, 3.94, and 11.82 mg/mL) in S-complete liquid medium with FUDR for 48 hours; the worms were then transferred to a 48-well plate, with approximately 35 worms in 160 *μ*L of M9 buffer per well [24]. Oxidative stress was induced with 10 mM hexavalent chromium (Cr [VI], K_2_Cr_2_O_7_, Sangon Biotech, Shanghai, China); before the stressor was added, the worms were washed three times with M9 buffer to remove OP50 bacteria. Worm survival was monitored regularly every 4 hours using the touch-provoked movement described above. The experiment was conducted in three independent repeats.

### 2.6. Measurement of ROS Production

The effect of GNC on ROS levels in* C. elegans* under oxidative stress conditions induced by Cr (VI) was measured using H_2_DCF-DA (Sigma-Aldrich Co., St. Louis, MO, USA) as a molecular probe. The procedures for GNC and Cr (VI) administration were the same as those outlined above for the oxidative stress resistance assay. The worms were washed three times with M9 buffer, and 50 *μ*M H_2_DCF-DA was added to each well. After incubating for 3 hours at 20°C, the worms were mounted onto microscope slides with 2% agar pads. The worms were anesthetized with 10 mM NaN_3_ and then analyzed using a Nikon SMZ 1500 fluorescence microscope at 480/40 nm excitation and 535/50 nm emission. Quantitative image analyses were performed by image-based morphometric analysis (NIS-Elements D3.1, Japan) and Image J software (US National Institutes of Health, Bethesda, MD, US). Inverted fluorescent images were used for the analysis. Positive signals were defined with Image J. At least 20 animals from each group were quantified, and the experiment was performed three times independently.

### 2.7. Quantitative Analysis of Stress-Induced Genes in* C. elegans*

Synchronized adult worms were exposed to Cr (VI) as described above for 15 hours at 20°C. The worms were collected, washed three times with M9 buffer, transferred to RNase-free microfuge tubes, and pelleted by centrifugation at 4,000 rpm for 1 min. The samples were freeze-thawed, and 1 mL TRIzol reagent (Thermo Fisher Scientific, Shanghai, China) was added to each sample. For RNA extraction, 200 *μ*L of chloroform was added, and the worm suspension was shaken vigorously and centrifuged at 12,000 ×g for 10 min. The total nematode RNA in the supernatant was isolated using isopropanol and washed with ethanol. The RNA concentration was quantified using a Nanodrop spectrophotometer. cDNA was synthesized by reverse transcription using FastQuant RT Kit (with gDNase; Tiangen, Beijing, China) according to the manufacturer's protocol. Quantitative real-time polymerase chain reaction (qPCR) was performed using qTOWER 2.2 Real-Time PCR System (Analytik Jena AG, Thuringia, Germany) with SuperReal PreMix Plus (SYBR Green; Tiangen, Beijing, China). The primers were as follows: sod-3, forward, 5′-AGCATCATGCCACCTACGTGA-3′, and reverse, 5′-CACCACCATTGAATTTCAGCG-3′; ctl-2, forward, 5′-TCCGTGACCCTATCCACTTC-3′, and reverse, 5′-TGGGATCCGTATCCATTCAT-3′; mtl-1, forward, 5′-ATGGCTTGCAAGTGTGACTGCAAAAACAAGC-3′, and reverse, 5′-TTAATGAGCCGCAGCAGTTCCCTGGTGTTGATGGG-3′; hsp-12.6, forward, 5′-GTGATGGCTGACGAAGGAAC-3′, and reverse, 5′-GGGAGGAAGTTATGGGCTTC-3′; and hsp-16.2, forward, 5′-CTGCAGAATCTCTCCATCTGAGTC-3′, and reverse, 5′-AGATTCGAAGCAACTGCACC-3′. The gene act-4 (forward: 5′-GCCACCGCTGCCTCCTCATC-3′ and reverse: 5′-CCGGCAGACTCCATACCCAAGAAG-3′) was used as a nonvariable control. Relative fold changes were calculated using the 2^−ΔΔCT^ method. The experiment was repeated in triplicate.

### 2.8. Statistical Analyses

GraphPad Prism 6.0 was used for statistical analyses. For the lifespan assay, Kaplan-Meier survival analysis was conducted, and *p* values were calculated using the log-rank test. Student's* t*-test was performed to compare two datasets. One-way analysis of variance (ANOVA) with Duncan's post hoc test was used to compare more than two datasets. All results were expressed as the means ± standard error of the mean (SEM). Values of *p* < 0.05 were considered significant.

## 3. Results

### 3.1. GNC Extended Wild-Type* C. elegans* N2 Lifespan under Normal Conditions

**Figure 1:**
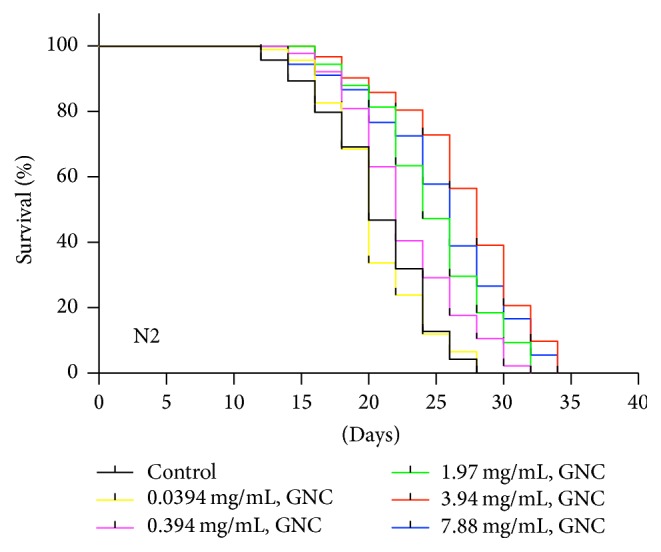
Effect of GNC on the lifespan of* C. elegans* N2 under normal conditions at 20°C. Notes: the above curves show the survival percentage of worms on different days after treatment with a vehicle control (H_2_O) or various doses of GNC (0.0394, 0.394, 1.97, 3.94, or 7.88 mg/mL). GNC increased the lifespan of wild-type worms in a dose-dependent manner. The maximum increase in lifespan was observed at a dose of 3.94 mg/mL GNC (*p* < 0.0001).

To evaluate whether GNC has prolongevity properties under normal conditions, we treated wild-type* C. elegans* N2 on the first day of adulthood with different concentrations of GNC at 20°C (we chose GNC doses of 0.0394, 0.394, 1.97, 3.94, or 7.88 mg/mL, which were found to be nontoxic to the worms (Supplementary Table  1 and Figure 1 in Supplementary Material available online at https://doi.org/10.1155/2017/8432306)). As shown in Figure 1, most doses (0.394, 1.97, 3.94, and 7.88 mg/mL) of GNC significantly increased the mean lifespan of adult worms (10.0% for 0.394 mg/mL, *p* = 0.0009; 21.0% for 1.97 mg/mL, *p* < 0.0001; 31.3% for 3.94 mg/mL, *p* < 0.0001; and 23.0% for 7.88 mg/mL, *p* < 0.0001) compared with the control. However, the dose of 0.0394 mg/mL did not lead to a significant extension of lifespan (*p* = 0.7742). These results show that 3.94 mg/mL provided the maximum lifespan increase.

### 3.2. GNC Treatment Increased Stress Resistance and Reduced ROS Levels under Oxidative Stress Conditions in* C. elegans*

**Figure 2:**
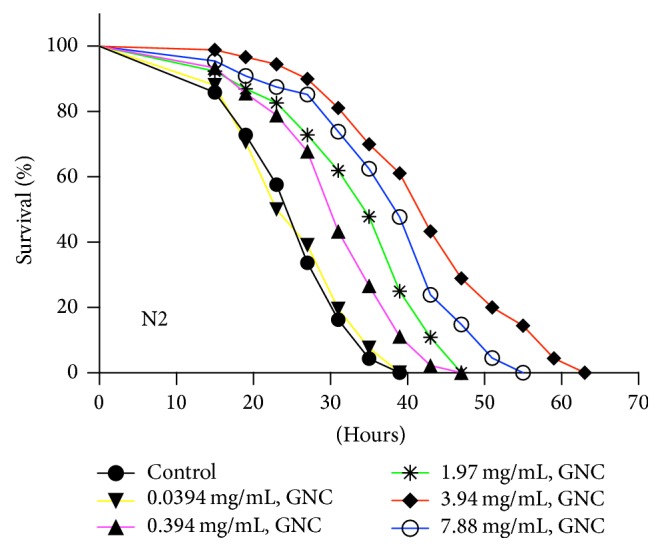
The effect of GNC on oxidative stress resistance in* C. elegans* N2. Notes: GNC (0.0394, 0.394, 1.97, 3.94, or 7.88 mg/mL) and vehicle control (H_2_O)-pretreated wild-type worms were exposed to 10 mM Cr (VI). Survival after each treatment was assessed every 4 hours. GNC increased the mean and maximum survival times of worms under oxidative stress conditions in a dose-dependent manner.

Numerous studies on* C. elegans* have suggested that enhanced stress resistance is an important factor associated with extending lifespan. To investigate whether GNC has an antioxidative effect,* C. elegans* N2 adults were pretreated with GNC (0.0394, 0.394, 1.97, 3.94, or 7.88 mg/mL) for 48 hours, followed by exposure to Cr (VI). Cr (VI) is a heavy metal with lethal toxicity that generates intracellular ROS, inducing oxidative stress. The results showed that pretreatment with 3.94 mg/mL GNC maximally increased the mean lifespan of wild-type* C. elegans* N2 under Cr (VI)-induced oxidative stress by 67.0% (43.13 ± 1.17, *p* < 0.0001) compared with the control (25.83 ± 0.71, Table 2). Other doses of GNC pretreatment had a similar antioxidative stress effect (0.394 mg/mL, 21.4%, *p* < 0.0001; 1.97 mg/mL, 32.5%, *p* < 0.0001; and 7.88 mg/mL, 48.9%, *p* < 0.0001), whereas a lower dose of GNC (0.0394 mg/mL, *p* = 0.8677) did not have a significant effect (Figure 2). These findings suggest that GNC enhances stress resistance in a dose-dependent manner. Given that the most significant increase in mean lifespan of wild-type N2 worms under normal or stress conditions was achieved at 3.94 mg/mL GNC, we chose this concentration to perform ensuing experiments.

**Figure 3:**
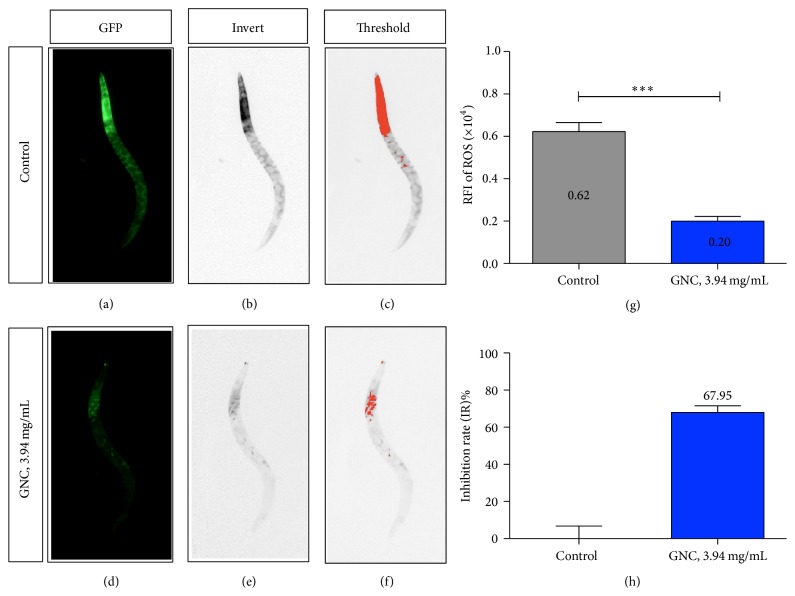
ROS levels of* C. elegans* under oxidative stress conditions. Notes: worms were exposed to Cr (VI) for 24 hours and stained with the molecular probe H_2_DCF-DA. Images of worms pretreated with the vehicle control (H_2_O) are displayed as follows: fluorescent image (a), inverted image (b), and positive signal (c). Worms pretreated with 3.94 mg/mL of GNC are displayed as follows: fluorescent image (d), inverted image (e), and positive signal (f). All images were characterized using Image J. Quantitative comparisons of the ROS levels depict ROS level reductions of 67.95% in GNC-pretreated worms (g, h). ^*∗∗∗*^*p* ≤ 0.001.

Next, we investigated whether GNC treatment decreased intracellular ROS levels under Cr (VI)-induced stress conditions. As presented in Figure 3, GNC (3.94 mg/mL) significantly reduced total ROS levels (67.95%, *p* < 0.0001) compared with the vehicle control, suggesting that GNC has ROS-scavenging ability.

### 3.3. GNC Increased Oxidative Stress Resistance in* C. elegans *via DAF-16/FOXO

**Figure 4:**
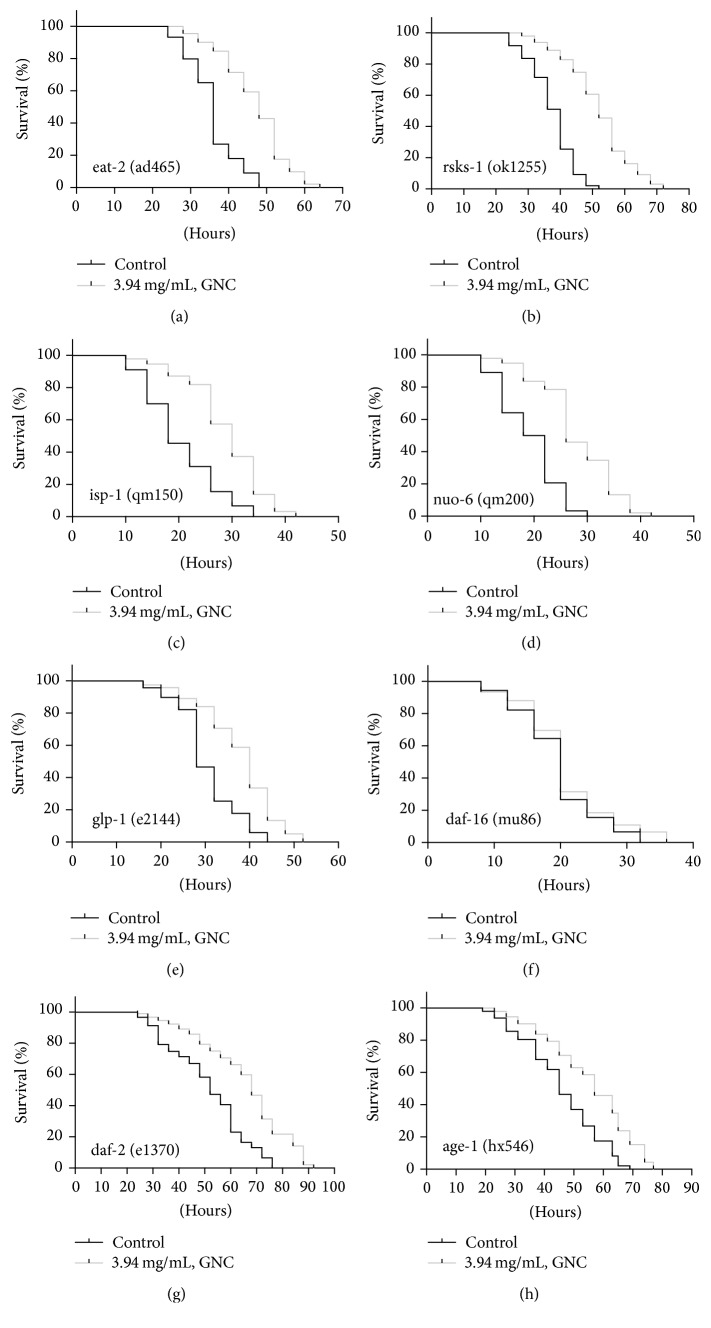
The effect of GNC on the oxidative stress resistance of characterized mutant strains. Notes: survival curves of mutants under Cr (VI)-induced oxidative stress conditions: (a) eat-2 (ad465); (b) rsks-1 (ok1255); (c) isp-1 (qm150); (d) nuo-6 (qm200); (e) glp-1 (e2144); (f) daf-16 (mu86); (g) daf-2 (e1370); and (h) age-1 (hx546), pretreated with an H_2_O control (black) or 3.94 mg/mL of GNC (grey). The protective effect of GNC depended on the function of daf-16 but was independent of the eat-2, rsks-1, isp-1, nuo-6, glp-1, daf-2, and age-1 genes.

Many genes and pathways are associated with oxidative stress responses and longevity (e.g., the IIS, dietary restriction (DR), and germline signaling pathways). To determine the genes/pathways involved in GNC-mediated increases in oxidative stress resistance, we performed Cr (VI)-induced oxidative stress tests using several characterized mutant strains. Our results showed that GNC did not enhance stress resistance in daf-16 mutants, indicating that daf-16 function is essential for the observed GNC-mediated increase in oxidative stress resistance. In contrast, significant lifespan enhancement was maintained in other mutant strains, daf-2 (e1370), age-1 (hx546), nuo-6 (qm200), isp-1 (qm150), eat-2 (ad465), rsks-1 (ok1255), and glp-1 (e2144) (Figure 4), suggesting that the protective effect of GNC is independent of the function of these genes.

**Figure 5:**
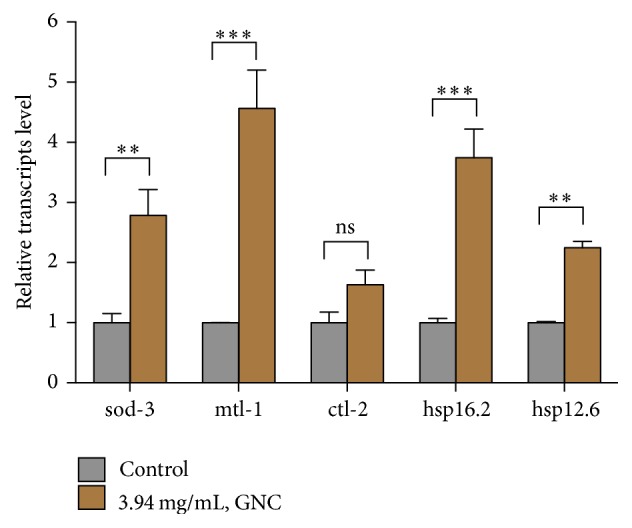
Quantification of mRNA expression levels of daf-16-targeted genes in GNC-treated worms. Notes: GNC (3.94 mg/mL) treatment affected relative transcript levels of the genes sod-3, mtl-1, ctl-2, hsp-12.6, and hsp-16.2. *β*-Actin (act-4) was used as an endogenous control, and the expression levels were determined by real-time PCR using the 2^−ΔΔCT^ method. The data are displayed as the means ± SEMs. ^*∗∗*^*p* ≤ 0.01; ^*∗∗∗*^*p* ≤ 0.001; ns: not significant.

To further confirm that GNC enhances oxidative stress resistance in* C. elegans* by regulating daf-16-encoded activity, we examined the effect of GNC on the following daf-16-targeted genes: sod-3, mtl-1, ctl-2, hsp-12.6, and hsp-16.2 [25, 26]. Expression levels of sod-3, mtl-1, hsp-12.6, and hsp-16.2 were significantly increased, and the upregulation of ctl-2 exhibited a tendency toward significance (1.6-fold, *p* = 0.068). These results suggest that GNC increases oxidative stress resistance via daf-16 (Figure 5).

## 4. Discussion

Traditional Chinese medicine is widely used to relieve declining functions related to aging [2, 27]. Several studies have shown that GNC improves learning and memory, delays skin aging, and confers resistance to stress conditions [5, 6]. However, the mechanism through which GNC exerts these actions remains unclear. The current study employed* C. elegans* as an in vivo model to investigate the protective potential and mechanisms of GNC. We observed significant lifespan increases of 21.0%, 31.3%, and 26.3% at 1.97, 3.94, and 11.82 mg/mL of GNC, respectively. These findings indicate that GNC extends the lifespan of* C. elegans* in a dose-dependent manner.

As previously reported, oxidative stress damage increases while resistance to cellular stress declines with age [28]. The enhanced lifespan of* C. elegans* has been correlated with improved oxidative stress resistance, and multiple studies have shown that lifespan-extending interventions affect resistance to oxidative stress [29, 30]. Accordingly, we performed an oxidative stress resistance assay, and our findings showed that the survival time of wild-type worms under oxidative stress conditions was significantly increased following GNC treatment. Moreover, we found that GNC reduced ROS levels by 67.95%, which suggests that GNC has antioxidative capacities due to its ROS-scavenging ability and further indicates that lifespan is positively correlated with resistance to oxidative stress. Although the exact role that oxidative stress plays in the aging process is unknown, it clearly is crucial. Furthermore, oxidative stress damages cellular structures and results in premature cell death. Increasing evidence shows that oxidative stress is involved in various diseases including neurological and cardiovascular diseases as well as metabolic disorders [14]. Previous research demonstrated that GNC has a protective function in oxidative-associated disorders such as skin aging in rat models [6], and given its antioxidative activity in* C. elegans*, we hypothesized that antioxidative activity is a primary mechanism of the effects of GNC.

Insulin/IGF-1 signaling, germline signaling, DR, and mitochondrial respiration have all been extensively studied, and these pathways have conserved roles not only in regulating the aging process but also in controlling other functions such as stress resistance, metabolism, and reproduction [31]. To determine which pathways are involved in the antioxidative activity of GNC, we examined its effect on nematode strains with mutations in these pathways.

DR increases stress resistance, and previous studies have shown that it induces defensive mechanisms involved in ROS detoxification [32] and increases resistance to lethal heat stress [33]. The eat-2 (ad465) II mutant is regarded as a DR model with pharyngeal pumping defects. In our study, the average survival time under the oxidative condition of eat-2 (ad465) II pretreated with GNC was extended by 32% (*p* < 0.001), indicating that GNC might not act through a DR mechanism.

mTOR signaling is a highly conserved pathway that regulates cellular stress responses, autophagy, and metabolism. TORC1 mediates phosphorylation of S6 kinase (S6K), thereby playing a major role in mRNA translation [34]; the putative ribosomal protein S6K is encoded by rsks-1. We tested survival time of mutant rsks-1 (ok1255) worms under oxidative stress and found that GNC increased antioxidative activity in rsks-1 mutants by up to 39% (*p* < 0.001), which suggests that GNC does not act on the mTOR signaling pathway.

Mitochondrial respiration plays a central role in energy metabolism; indeed, the ROS produced within mitochondria represent almost 90% of all cellular ROS produced [35]. To investigate whether GNC increases the antioxidative activity of* C. elegans* via the mitochondrial respiration pathway, we tested the effect of GNC on the mutants isp-1 (qm150) and nuo-6 (qm200), encoding Rieske iron-sulfur protein in complex III and a subunit of the NADH dehydrogenase complex [36], respectively. The results showed that GNC increased average survival times under oxidative conditions in isp-1 and nuo-6 (qm200) worms by 42% (*p* < 0.001) and 47% (*p* < 0.001), respectively. These results suggest that the antioxidative activity of GNC does not depend on the isp-1 and nuo-6 genes.

In addition to propagation, the reproductive system of* C. elegans* is involved in regulating metabolism, autophagy, stress resistance, and longevity [37]. We performed an oxidative stress resistance assay using the mutant glp-1 (e2144) and found increases in antioxidative activity of 24% (*p* < 0.001) by GNC, suggesting that GNC functions independently of the germline signaling pathway.

The FOXO transcription factor daf-16 plays a central role in regulating the cellular stress response and promoting cellular antioxidant defenses [25]. We tested whether daf-16 is required for the protective effect of GNC, and our results showed that GNC did not increase the survival time of null mutant daf-16 (mu86) worms (*p* > 0.05) under oxidative stress conditions. This finding suggests that the effect of GNC depends on daf-16. To further validate that GNC increased antioxidative activity in worms by regulating daf-16 activity, we measured the expression levels of the following daf-16-targeted genes: sod-3, mtl-1, ctl-2, hsp16.2, and hsp12.6. Levels of sod-3, mtl-1, hsp16.2, and hsp12.6 were all significantly increased in worms pretreated with GNC, suggesting that GNC mediates oxidative stress resistance via daf-16. Insulin/IGF-1 signaling, the first pathway discovered to be associated with lifespan, ultimately regulates the forkhead transcription factor daf-16 [38]; daf-2, an ortholog of the mammalian insulin and insulin-like growth factor-1 (IGF-1) receptor, phosphorylates age-1, the catalytic subunit of class-I phosphatidylinositol 3-kinase (PI3K), which normally generates PIP3, thereby preventing daf-16 from activating via stimulation of Akt (AKT-1/2) [39]. To investigate whether GNC interacts with molecules in the insulin/IGF-1 signaling pathway to increase antioxidative functions and daf-16 activity, an oxidative stress resistance assay was conducted using mutant daf-2 (e1370) and age-1 (hx546) strains. The results showed that GNC increased average survival time in daf-2 (e1370) and age-1 (hx546) by 28% (*p* < 0.001) and 21% (*p* < 0.001), respectively, under oxidative conditions. Thus, GNC induces oxidative stress resistance independent of the insulin/IGF-1 signaling pathway. Taken together, our data suggest that GNC enhances oxidative stress resistance via daf-16/FOXO in* C. elegans*. Daf-16/FOXO integrates signals from multiple pathways in* C. elegans*, regulating many important biological processes including lifespan, development, metabolism, and stress resistance. However, previous research has indicated that daf-16 does not function alone; rather, it requires other molecules for its activity. c-Jun N-terminal kinase (JNK), a stress-activated MAPK family member, phosphorylates daf-16 in vitro, acting in parallel with the insulin-signaling pathway to regulate daf-16 directly. Similarly, CST-1, the* C. elegans* homolog of the mammalian Ste20-like kinase MST1, regulates daf-16 in response to oxidative stress [40]. When activated, daf-16 relocates to the nucleus and stimulates transcription of genes that encode antioxidant proteins including antioxidant enzymes such as superoxide dismutase (sod-3), catalases (ctl-1, ctl-2), and metallothionein (mtl-1) [25]. Moreover, together with HSF-1, daf-16 promotes expression of the small heat-shock protein genes hsp-16.1, hsp-16.49, hsp-12.6, and sip-1 [41]. Daf-16 also regulates transcription of numerous other genes to protect worms from damage due to heat stress, oxidative stress, and pathogens, thereby increasing survival under harsh conditions. Furthermore, daf-16 is involved in posttranslational modifications and interactions with coregulators that constitute important transcriptional regulatory mechanisms [25].

A wide variety of modern aging-related diseases such as cancer, diabetes, and neuronal degeneration diseases have become significant threats to human health, and importantly, susceptibility to these diseases increases with age [42]. As opposed to more conventional, single-disease treatment approaches, application of TCM formulas to interfere with the aging process to delay the onset of age-related diseases has begun to receive more attention. However, research on the nature of TCM formulas is rather difficult due to the composition complexity. The results of the present study strongly indicate that the GNC formula enhances resistance to oxidative stress and extends lifespan. It will be interesting and important to determine the ingredient(s) responsible for these effects of GNC in future studies.

## 5. Conclusions

In summary, the current study reports that GNC, a TCM, increases resistance to oxidative stress and promotes longevity in* C. elegans*. These findings are consistent with those of recent studies showing that GNC protects against oxidative stress and has beneficial effects against age-related diseases [3–6]. The antioxidative stress activity of GNC depends on daf-16/FOXO and selective activation of its downstream targets associated with oxidative stress defenses. These findings suggest that GNC acts as an antioxidative agent, and regulation by daf-16 partially explains the therapeutic effects of GNC. Therefore, GNC shows therapeutic potential for preventing oxidative stress-related diseases such as Alzheimer's disease. Additional tests should be conducted using more complex animals. In addition, because TCM has an expansive pharmacopeia with an array of biological activities that are varied and complex, our use of* C. elegans* as an animal model to investigate the mechanism of GNC provides a novel method for screening the biological mechanisms of TCMs.

## Supplementary Material

To evaluate whether GNC was toxic to the worms, we conducted the toxicity test for GNC. As shown in Supplementary Table 1; Figure 1, the doses of 0.00394, 0.0394, 0.394, 3.94, 7.88 mg/mL were found to be nontoxic to the worms. However, the dose of 15.76, 39.4, 78.8 mg/mL lead a survival rate 60%~83%, indicating these doses were toxic to the worms.

## Figures and Tables

**Table 1:** Composition and preparation of GNC.

TCM ID	Crude herb (g)	Water extract (g)
Radix Rehmanniae	15	4.5
Rhizoma Coptidis	3	0.5
Radix Paeoniae Alba	12	1.2
Rhizoma Anemarrhenae	15	3.75
*Cistanche salsa*	12	3.6
Radix Morindae Officinalis	12	3.6
Poria	9	0.9
*Epimedium brevicornum*	12	0.6
Cortex Phellodendri Amurensis	9	0.75
*Fructus lycii *	12	4.8
Semen Cuscutae	12	0.6
Carapax et Plastrum Testudinis	15	0.75

Notes: conversion between crude herb and water extract: for example, 4.5 g water extract of Radix Rehmanniae is equivalent to 15 g crude herb.

**Table 2:** Survival time data under oxidative stress: effects of GNC treatment on wild-type and mutant *C. elegans*.

Strain	Treatment (mg/mL)	Mean survival hours (±SEM)	Worms (*N*)	% change	*p* value
N2	Control	25.83 ± 0.71	92	67	<0.0001
	3.94-GNC	43.13 ± 1.17	90		
eat-2 (ad465)	Control	35.69 ± 0.68	89	32	<0.0001
	3.94-GNC	46.95 ± 0.90	91		
rsks-1 (ok1255)	Control	37.39 ± 0.69	98	39	<0.0001
	3.94-GNC	51.88 ± 1.02	99		
isp-1 (qm150)	Control	20.40 ± 0.71	90	42	<0.0001
	3.94-GNC	28.94 ± 0.72	94		
nuo-6 (qm200)	Control	19.09 ± 0.57	92	47	<0.0001
	3.94-GNC	28.04 ± 0.72	98		
glp-1 (e2144)	Control	30.54 ± 0.62	95	24	<0.0001
	3.94-GNC	37.92 ± 0.76	96		
daf-16 (mu86)	Control	19.60 ± 0.63	90	0.06	0.2311
	3.94-GNC	20.74 ± 0.70	92		
daf-2 (e1370)	Control	51.43 ± 1.56	91	28	<0.0001
	3.94-GNC	65.87 ± 1.76	92		
age-1 (hx546)	Control	45.82 ± 1.31	97	21	<0.0001
	3.94-GNC	55.61 ± 1.51	92		
