# Supplementary material for: Gengnianchun, a Traditional Chinese Medicine, Enhances Oxidative Stress Resistance and Lifespan in Caenorhabditis elegans by Modulating daf-16/FOXO
Source: Evid Based Complement Alternat Med. 2017 Mar 16;2017:8432306. doi: 10.1155/2017/8432306 (PMC5376401; doi:10.1155/2017/8432306)
Supplement: Supplementary file 1 — To evaluate whether GNC was toxic to the worms, we conducted the toxicity test for GNC. As shown in Supplementary Table 1; Figure 1, the doses of 0.00394, 0.0394, 0.394, 3.94, 7.88 mg/mL were found to be nontoxic to the worms. However, the dose of 15.76, 39.4, 78.8 mg/mL lead a survival rate 60%~83%, indicating these doses were toxic to the worms. [file 8432306.f1.pdf]

Table1: Toxicity exposed to different concentrations of GNC

GNC (mg /mL)	0.00394	0.0394	0.394	3.94	7.88	15.76	39.4	78.8
Dead (N)	0	0	0	0	0	15	24	36

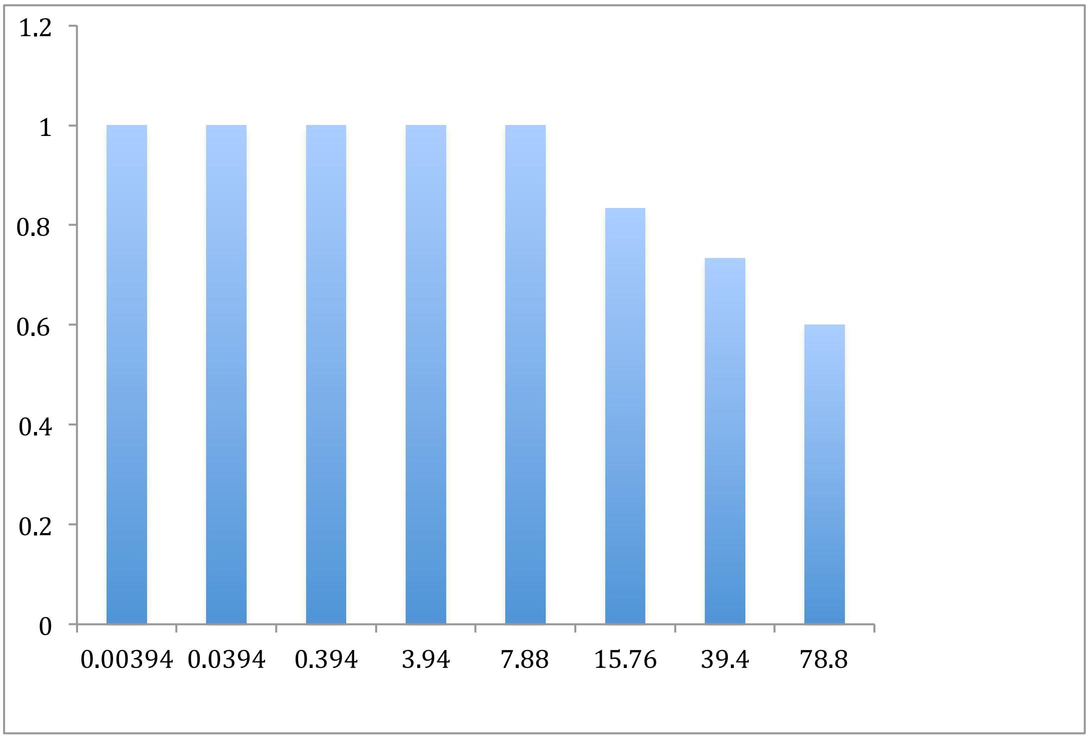

Fig 1: Survival percentage of worms after 48 hours treated with various concentrations of GNC (mg/mL); 90 worms for each concentration.
